# Factors promoting and hindering sporting success among South African former Olympians from historically disadvantaged areas

**DOI:** 10.17159/2078-516X/2023/v35i1a15068

**Published:** 2023-04-13

**Authors:** S Mthombeni, Y Coopoo, H Noorbhai

**Affiliations:** Department of Sport and Movement Studies, Faculty of Health Sciences, University of Johannesburg, Johannesburg, South Africa

**Keywords:** support systems, historically disadvantaged areas, Olympics, athletes

## Abstract

There are various contributing factors to sporting success among elite athletes, including Olympians. The purpose of this paper was to investigate the enablers and/or barriers to sporting success among South African former Olympians from historically disadvantaged areas (HDAs) using the SPLISS framework. This would enable an understanding of the factors that lead to sporting success among athletes from HDAs. A qualitative research design was employed for this study, whereby semi-structured interviews were conducted among 15 former Olympians who represented South Africa between the 1992 and 2016 Olympic Games. The ATLAS.ti (version 22) software tool was used to analyse the data. The study found that athletes from HDAs attributed their sporting success to the functional competition structure, sports access at community level, access to scholarships and bursaries to elite schools/universities, good coaching support and mentorship, access to local and international competitions, as well as community and peer athlete support. The highest barriers reported by athletes were inadequate financial support, a dysfunctional school sport system, lack of sports facilities, equipment and transport system, poor post-career and scientific support. Elite athletes from HDAs need consistent financial support, school/foundation level sport access, quality sports facilities, equipment, and reliable transport to training and competitions, post-career, as well as scientific support to achieve their full potential and attain international sporting success.

International sporting success in any nation is centred on achieving international prestige, socioeconomic development, and enhancement of national pride, to name a few. Therefore, increasing competitiveness for sporting success encouraged global investments towards elite sports policies among nations to facilitate elite sport development through a uniform model. More importantly, the success of an individual athlete or team is dependent on the national system's performance capacity and its effectiveness in the usage of resources toward elite sporting success. ^[1]^ De Bosscher et al. ^[1]^ developed a conceptual framework of sport policy factors leading to sport success (SPLISS) that would be used as a model for nations to assess sporting success. The SPLISS model consists of nine pillars including financial resources, governance, organisation and sport policy, foundation level participation, identification and development of talent, post-sport career support, provision for sports infrastructure, provision for coaching and development, access to national and international competitions, as well as scientific research and support. ^[1]^ De Bosscher et al. ^[1]^ used the mixed methods approach for SPLISS to compare policies of elite sport among 15 countries and indicated that the composite indicators were helpful in the identification of a relationship or non-relationship between sports policies and success, facilitation of comparison and interpretation of counties, as well as the understanding of differences in elite sport systems. Mazzei et al. ^[2]^ evaluated the structure of the Brazilian high performance sports policies through the SPLISS framework and found the existence of financial resources, but a lack of strategic planning and integration of the sports policies, leading to ineffective management of the application of resources. Therefore, more financial resource allocations towards high performance sport does not necessarily lead to a better international performance in sport if the other pillars of support are not well structured. A better synergy between the pillars of support is necessary for international sporting success. ^[2]^

In the South African context, sport played a central role in transforming South Africa by creating a post-apartheid identity that was aimed at eliminating divisions on a basis of race, gender, class, and geographical location. ^[3]^ Apartheid was a South African policy and ideology of racial segregation. ^[3]^ The National Sport and Recreation Plan (NSRP) ^[4]^ highlighted that the system of apartheid led to gross inequality, whereby over 50% of the South African population, which were predominantly black Africans, lived below the poverty line. This led to inequalities and an uneven playing field within the sports sector. ^[4]^ The post-apartheid government elected in 1994 developed the new Department of Sport and Recreation South Africa (SRSA), now referred to as the Department of Sport, Arts, and Culture (DSAC), as the government department the mandate of which was, largely, to address the injustices of the past by ensuring equality in the field of sport and establishing the Transformation Charter. ^[4]^ Coetzee et al. ^[5]^ reported that racial inequalities in sports from the apartheid era continue to affect sports participation or non-participation on a basis of race, across various sporting codes in South Africa, as different opportunities for most sports are still not accessible for the black population. Desai ^[6]^ argues that although the sport of swimming in South Africa, for instance, continues to claim global dominance in terms of competitiveness, it is still primarily regarded as a ‘white’ sport as it remains dominated by swimmers from the white community, more especially at the highest level of competition. This is a consequence of the sport infrastructure (especially swimming facilities) being historically concentrated in the recreational and residential areas of the white community during apartheid, and which were legislated to only be used by the white population. Recreational facilities, including functional swimming facilities were inadequate or non-existent in the predominantly black townships and the effects continued to be felt in post-apartheid South Africa, making it almost impossible to nurture talent from the black townships. ^[6]^ The Transformation Charter defines transformation as a process of changing the delivery of sport holistically through the actions of individuals and organisations to harness the socioeconomic benefits of sport. The Charter aimed at increasing access and opportunities in sport and recreation for all citizens, with particular emphasis on previously disadvantaged groups, including but not limited to, black (i.e. African, Coloured, and Indian) population groups which included, women, children, youth, persons with disabilities, as well as the elderly. ^[5]^ Therefore, historically disadvantaged areas (HDAs) in this study referred to areas that were underfunded and underdeveloped during the apartheid era, whereby the legacy of underdevelopment persists to date. These areas include townships, farms, and rural areas (as well as villages). ^[7]^ Xaba and Malindi ^[7]^ highlight HDAs as historical settlements characterised by poor socioeconomic conditions, including poor infrastructure development, and most were designated as living areas for black people. Dove et al. ^[8]^ investigated career progression to the elite level among black South African cricket players and found that game exposure, coaching, facilities and equipment, education, and support networks as enablers of success, and poor socioeconomic factors, development pathways, team environment and leadership were barriers to success. This required a need to further investigate whether other sporting codes, or sport in general, has similar enablers and barriers to success in South Africa, thus necessitating a need for further insight into potential success factors and barriers to sporting success, with a particular emphasis on athletes from HDAs. The most important research questions for this study were as follows: Do the current systems of sport support in South Africa create an enabling environment for an athlete to achieve international success? If so, what are these enabling factors, and if not, what are the hindrances?

Sport in South Africa is under the responsibility of the national Department of Sport, Arts, and Culture (DSAC), which has mandated the South African Sports Federation and Olympic Committee (SASCOC), along with the national sports federations (NSFs) to deliver high performance sport in South Africa to all. Tertiary institutions, sports academies, and professional sports clubs are structures that also form part of the pathway for elite athletes to reach high performance in sports. ^[4]^ The figure above shows a typical distribution mechanism for the delivery of high performance sports in South Africa. Figure 1 illustrates the South African support mechanism for high performance sport.

The purpose of the paper is to investigate the enablers and/or barriers to sporting success among South African former Olympians from HDAs using the SPLISS framework. This would enable an understanding of the factors that lead to sporting success among athletes from HDAs, and overcoming barriers to sport participation.

## Methods

### Study design and participants

A qualitative research design was employed for this study. A total of 15 athletes (13 males and 2 females) participated in semi-structured interviews as part of the study procedure (Table 1). The participants in the study were athletes that have represented South Africa at the Olympic Games between 1996 and 2016. The participants were current and retired South African athletes that were born and had resided in HDAs at some stage of their sporting careers. The participants were all black African former Olympians between the ages of 29 and 50 years, of which nine were still active athletes and six had retired from their sport. The sporting codes represented included: athletics (n=7), rugby sevens (n=2), boxing (n=2), football (n=1), canoeing (n=1), hockey (n=1), and sailing (n=1).

### Ethical considerations

The Faculty of Health Sciences Research Ethics Committee at the University of Johannesburg provided ethical approval for the study (REC-500–2020). The participants provided consent prior to participating in the study.

### Data collection

The interviews were conducted telephonically and audiotaped. A portable Tablet voice recorder (Lenovo Tab M10 HD, Slovakia) was used for the recording of the interviews with participants. The interviews were, on average, 38 minutes in duration. A step-by-step interview guide was used to ensure that the interviews occurred similarly and systematically. All nine interview questions were open-ended and were in line with the SPLISS framework, with questions investigating enablers in, as well as barriers to, elite sport participation under the following themes; access to financial resources; governance, organisation, sports policies, access to competitions; opportunities for sport participation at foundation level; talent scouting and development; post-career support; access to sports facilities; access to coach support and development; access to international competitions; and access to scientific support (Table 2). Follow-up questions were also conducted to allow participants to provide further insight into the matters discussed.

### Data analysis

The interview data were translated from the audio recorder and transferred to a Laptop for storage and analysis. The audio data were then transcribed manually and through a computer software known as Otter.ai (Los Altos, California, US) into text format by the interviewer. Upon the conclusion of the transcribing, all the text captured in the native language of the participants was translated into English. Familiarisation of the data was conducted to understand the breadth and depth of the content. A thematic analysis approach was employed. The initial codes were formed based on the interesting concepts to analyse. From the initial codes, a total of 42 meaningful themes that highlighted the athletes’ career pathways and experiences were identified. The themes were then categorised into 11 factors of sport support. From the 11 factors of sport support, six enablers and five barriers to sporting success were identified, followed by the write-up of the findings. The ATLAS.ti (version 22) software tool was used to analyse the data.

## Results and Discussion

The results of the study found that factors that enabled sporting success among athletes from HDAs were functional sport structures, access to community sport, bursaries and scholarships, coaching support and mentorship, local and international competitions, as well as community and peer support. Factors that were found to hinder sporting success among athletes from HDAs were lack of financial support, a dysfunctional school sport system, lack of facilities and equipment, lack of post-career support, and poor scientific support.

### Enablers

#### Functional sport structures

**Fig. 2:**
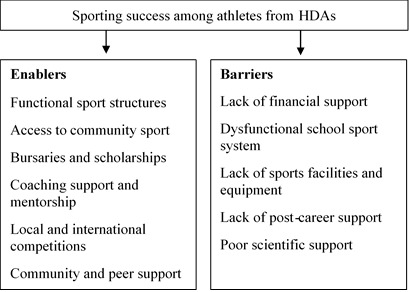
Results showing the perceived enablers and barriers in South African sport by athletes

When asked about governance, organisation and sport policy, the majority of athletes reported that South Africa has functional sports structures which provide an enabling environment to compete in sport from local, progressing to regional, provincial and national championships. International colours were provided to them when performing well at a national level. The athletes also reported that there are sufficient local league competitions for them to compete in and gain recognition (Figure 2). One commented as follows:

*‘We would compete in teams around our region, then provincial, then to National. The structures of competition and league system are good.’ (Athlete 9)*

The availability of governance, organisation and structure of sport policy was supported by Jacobs, De Bosscher, and Venter ^[9]^ who also reported on the availability of national, provincial, and local sports structures (government departments, local municipalities, and federations), all of which administer sport in South Africa as guided by the national White Paper on Sport and Recreation. However, it has been highlighted that interorganisational relationships between governmental sport stakeholders need to be further strengthened to allow for the understanding of the different roles and responsibilities that each entity needs to play in the field of sport. This will ensure that there’s no duplication of roles and the effectiveness of the delivery of sport programmes is improved (Jacobs, De Bosscher, and Venter. ^[9]^). Functional sport structures allow for athletes to compete regularly, and in a coordinated manner that allows those that are more talented to progress through the competition ranks from local to national, and eventually international level.

#### Access to community sport

The majority of athletes reported that one of the important factors that enabled them to participate in their respective sporting codes was that it was accessible within their community from a young age. It was reported that they participated in their local community sports clubs which offered the sport at a social and competitive level. However, most of the athletes also indicated that the sport was not accessible at the school level as their schools did not offer any structured sports programme. Few athletes had access to their sport at the school they attended, but they were able to attend well-resourced schools in suburban areas, not within their communities. Most of these opportunities came in the form of sports and academic scholarships. One was quoted as follows:

*‘…but for us in our community, the sport of canoeing was the main sport due to our proximity of the river nearby’ (Athlete 14)*

This finding was similar to that of Dove et al. ^[8]^ who found that early exposure to sport is one of the enabling factors for cricket success among South African black cricketers from townships and rural areas. This presents a challenge for South Africa, as physical education in predominantly disadvantaged schools does not form part of the curriculum and is merely seen as a voluntary extracurricular activity. Therefore, in most cases, athletes have to rely on volunteer-based local community sports clubs to get exposure and participation in sport. Attending wealthier elite semi-private or private schools with sufficient sporting infrastructure and support also comes with a distinct advantage as they serve as a feeder system for professional and national teams. ^[10]^ Noorbhai ^[10]^ found that the majority of the South African cricketers who played for the national team (the Proteas), mostly attended the wealthy elite and boys-only schools, irrespective of race. So in essence, athletes from HDAs who are unable to receive scholarships from these elite schools, may have to rely on local community sport clubs to navigate through sport, which in most cases, may be a more difficult route for the athlete, as most of the clubs may not be part of the feeder system to professional or national teams if cricket was used as an example. In the absence of physical education in low income schools, more local community-based clubs have to be established, and receive financial incentives from the government and the national lottery for developing athletes in the townships. This may indicate that government may need to reprioritise its spending and consider funding township-based sports clubs based on how many athletes they develop, more especially in unpopular sporting codes.

#### Bursaries and scholarships

All the athletes in this study reported being offered and provided with sports bursaries and scholarships by universities at some point in their sporting careers as part of their post-career support. Scholarships and bursaries in this study were two-fold, with some athletes provided with sports and academic scholarships to attend elite former model C schools (previous and current well-resourced semi-private public schools for the wealthier elite population group) in town, as well as university sports bursaries to fund their tertiary education studies. The bursaries not only allowed them to have access to education but also allowed them to be able to afford the necessities they needed for their sport (i.e. accommodation, sports attire, equipment, and nutrition). Athletes that went to former model C schools on scholarships had access to good sports infrastructure, school accommodation, nutrition, coaching and mentorship, and other support structures from an early age. However, to have access to such bursaries, they also needed to produce satisfactory academic results for admission into these elite schools or universities. One commented as follows:

*‘There were also opportunities for me as a senior athlete to have access to sports bursaries.’ (Athlete 4)*

The findings are supported by Thompson, Rongen, Cowburn, and Till, ^[11]^ who found that scholar athletes from sport-focused schools receive considerably more support in terms of academics, high-level training and competitions, recognition systems, and prospects of higher education continuation. These support systems positively contribute to the athletes’ long-term holistic development and success as an athlete when compared to their peer athletes who attended non-sport schools. Universities in South Africa offer varsity sports tournaments in most sporting codes. These tournaments serve as catalysts for student athletes to access high-level competition, financial and non-financial incentives and rewards, social well-being, as well as the promotion of their sporting career ^[12]^. This indicates that universities may present athletes from HDAs with opportunities for participation in sports that may not be available in the communities where they grew up. Bursaries also provide an opportunity for personal development of these athletes.

#### Coaching support and mentorship

The majority of athletes had access to coaching support from development (early age) until a high performance level. The athletes reported that although most of their developmental coaches were community volunteers, they all had the appropriate knowledge, skills, and coaching experience within the sport. Most athletes also highlighted that their coaches were their greatest mentors that propelled them toward sporting success. One profound comment was as follows:

*‘…one thing that made him stand out was that he understood where we came from as players, he knew that we came from under-resourced communities, so what made the relationship good was that he was able to mentor us despite the difficulties that we faced before in life.’ (Athlete 11)*.

Trzaskoma-Bicsérdy et al. ^[13]^ highlight that the nature of an athlete-coach relationship is more likely to determine the athlete’s performance, self-esteem, and satisfaction. When a coach can create an environment that fuels inspiration to the athlete and emphasises effort, the athlete is then able to positively respond by striving for excellence ^[13]^ Athletes in HDAs need to have access to an inspirational coach who is fully committed to supporting the athlete, both on and off the field of play. At times, athletes may lack parental figures who can provide moral support when playing sports, and the coach is therefore compelled to fill this important role. Hallmann, Breuer, and Beermann ^[14]^ defined a mentor as an experienced or high-ranking member in society or an organisation forming a relationship by coaching, advocating, and sponsoring the development of a less experienced mentee. Benefits of mentorship include increased personal development, satisfaction, self-worth, reputation and required skills, all of which are important for athletic success. ^[14]^ Therefore, it is important for coaching training and development programmes at federation level to include courses on mentorship and athlete welfare, so that they know how to provide the best possible support for their athletes.

#### Local and international competitions

The majority of athletes reported that some of their enablers towards sporting success included access to managers, agents, and professional clubs, which negotiated on their behalf and assisted them to compete internationally and gain international recognition. Some athletes also highlighted that the local competition structure was so highly competitive that it adequately prepared them for international success. Some commented as follows:

*‘I did have access to international competitions.’ ‘The more events that you compete in and win internationally, the more opportunities come to you to compete internationally.’ (Athlete 4)*

*‘Our competition system here in SA is good and there are enough competitions in the country, whether you have a sponsor or no sponsor.’ (Athlete 3)*

Ferguson ^[15]^ highlights that constant competitions, effective coaching, and access to appropriate sports facilities are among the most important factors for elite sporting performance and the success of nations. It is important, however, for athletes to have access to sufficient competitions from an early age as they progress in sporting performance and maturity. It is also important for South African national federations to produce quality local competition structures to enable athletes to progress through the competitive ranks, aiming for international success. Ferguson ^[15]^ further reiterates that high-quality local competitions allow athletes to test themselves against local competitors, and adequately prepares them for international success. De Bosscher, Du Bois, and Heyndeld ^[16]^ reported that an increase and exposure to the international competition environment increases competitiveness in athletes, and subsequently improvement in athletic performance. This, therefore, suggests that as athletes become more exposed to international competitions and compete at a higher intensity, they are more inclined to improve in their competitiveness and experience.

#### Community and peer support

The majority of athletes indicated that one of their enablers for sporting success was the support they received from their communities, including friends, family, community leaders, sports legends from their communities, as well as educators, all of whom were their key motivators. They also highlighted that it was through the volunteers and coaches within their communities who established local sports clubs, that enabled them to start participating in sports. Athletes also highlighted the importance of peer-athlete support in terms of learning and sharing of ideas on training regimes and techniques, sharing of sports equipment/attire and transport to competitions, as well as moral support being the important contributors of their success in sport. One commented as follows:

*‘There was a lot of support from my fellow boxers, including my brother, which played an important role in creating an environment for me to excel in the sport and become successful.’ (Athlete 9)*

This finding was supported by Shang and Yang ^[17]^ who found that social support had a positive impact in Chinese weightlifters by improving athlete motivation, mental toughness, and reducing burnout. Salcinovic et al. ^[18]^ also highlighted that supportive team behaviour is one of the key contributing factors to the success of high performance sports teams. Social support from teammates, coaches and support staff, family, and friends may present a positive relationship with the athlete's emotional, cognitive, and behavioural aspects, as well as their overall performance outcomes. ^[18]^

### Barriers

#### Lack of financial support

The majority of athletes reported a lack of financial support, especially at the early stages of their careers in sports. Most of the participants highlighted this as an obstacle, as they came from households where money was a scarce resource and their parents could only provide basic needs, while their sporting needs were regarded as luxuries they couldn't afford. At times, athletes who perform well and get selected to compete at national and international competitions were expected to personally fund their way on tours, as most of their families could not afford this, leading to low morale and discouragement. Some athletes also highlighted that sports clubs in townships had no sponsorship and were poorly funded, therefore inhibiting them to provide full support to the athletes in need (Figure 2). One athlete commented as follows:

*‘For us as black people, it's difficult as your still in the development page of the sport as there is very limited financial support that allows you to excel and remain in the sport.’ (Athlete 4)*

This finding was supported by Swart, Swanepoel, and Surujlal ^[19]^ who evaluated the South African government expenditure towards the sport and recreation sector and found that the majority of the budget allocation was towards office administration and minimal towards grass roots sports development and school sport. Jacobs, De Bosscher, and Venter ^[9]^ also found that the South African government allocated low grants towards elite sport development which could not meet the demands of the sector, with the national Olympic body of South Africa indicating that it heavily relies on sponsorships to fund its sport programmes. ^[9]^ This lack of investment by government eventually compromises the country’s effort in developing talented athletes. Financial investment in elite sport creates an enabling environment for athletes to attain international success. ^[2]^

One of the other challenging aspects that athletes also reported was having access to sponsorship support at the late stages of their careers. This presents a challenge for the period when the athlete is still at the ‘up-and-coming’ stage of their career. Hong and Fraser ^[14]^ highlight that elite athletes not funded by their national Olympic bodies, federations, or sponsors, are most likely to rely on family (mainly parents) and social networks of friends and relatives for financial support in some of the most crucial stages of their sporting journey. Athletes from well-resourced households may be able to receive better financial support from their families, whereas those from lower income households may not have the financial means to continue their sporting careers as a consequence of increased financial demands. This may be the reason for the high dropout rates of talented athletes from sports. Sports federations in partnership with government and sponsors should establish athlete-centred support programmes that are aimed at identifying talented athletes from marginalised communities, who show potential and provide the necessary financial and non-financial tools to ensure that they are well supported until they achieve financial independence.

#### Dysfunctional school sport system

Most of the athletes reported the dysfunctionality of the school sport system for schools in townships and rural areas as among the greatest obstacles to sports success. It was indicated that most of the township and rural schools do not offer any sport, nor do they have any facilities or equipment, and in some cases, only netball and soccer were the only competitive sporting codes on offer. It was also noted that educators in such schools have no keen interest in sports or encouraging sports participation. A few athletes reported having to migrate to elite schools in town through scholarships to have access to adequate sports infrastructure and support. One commented as follows:

*‘It is very concerning that a talented athlete from a township or a village has to be taken from the area to town to attend the elite schools to receive the necessary support.’ (Athlete 2)*

This finding was supported by the finding by Kanters et al. ^[20]^ who attributed the lack of sports programmes in low-income and predominantly black schools to funding reductions leading to other competing interests (academics demands), poor school sports policies, and exclusive school sports policy which limit sports participation in these schools. Noorbhai ^[10]^ also indicated that attending an elite (high-income) boys-only school was a strategic contributing factor toward cricket success among South African national cricket players. Therefore, it remains a concern that talented athletes from low-income households may need to have access to scholarships to attend elite schools in order to increase their probability of success in most sporting codes. The dysfunctional school sports system, coupled with poverty and inequality, was also highlighted as one of the main impediments to transformation, access, and equal opportunities within South African sport. The EPG transformation report also cited that as few as 2000 former model C public schools have formalised sports programmes. ^[21]^

#### Lack of sports facilities and equipment

The majority of athletes reported another constraint as a lack of access to professional sporting facilities within their area of residence, especially in the development phase. Some athletes reported making use of gravel and grass as their training grounds, or dams with slow water flow for canoeing, for instance. Many reported having access to good facilities after achieving elite status. The problem with poor sports facilities is that they affect the longevity of sports equipment, as the equipment is not designed for such terrains (i.e. athletic spikes on a gravel track instead of rubber). This then forces athletes to continuously replace equipment that they cannot afford. Some athletes reported having a lack of access to sports equipment throughout the early stages of their career, which led to them loaning from other athletes, and sports clubs, seeking donations of used equipment or sponsorship. One commented as follows:

*‘There are no good tracks in the township nor are there gym facilities, so in most cases, we relied on the tar road or gravel road for training.’ ‘We also trained on the gravel track and we did not complain because it made us stronger.’ (Athlete 4)*

Some of the athletes reported well-maintained sports training facilities being far from their residential areas, making transportation costs expensive. The athletes also highlighted that most competitions take place in the cities because of a lack of well-maintained and safe sporting facilities in townships and rural areas. This forces them to travel long distances using unreliable and expensive public transport system to be able to train and compete. One commented as follows:

*‘For some events, it was more difficult to get to competitions.’ ‘It's still difficult to reach competitions from there until today as most of the events are held in the Cities, which are about three hours away from my village.’ (Athlete 5)*

The lack of sports facilities in HDAs can largely contribute to the legacy of apartheid whereby predominantly black residential areas were largely underdeveloped in terms of infrastructure, with sports infrastructure forming part of the underdevelopment. ^[7]^ The problem of the lack of facilities is not only in communities but also in public schools. The South African National Education Infrastructure Management System Report of 2021 found that 10 038 out of 23 276 (43%) South African public schools have no sports facilities. Among 80% of these schools are no-fee paying schools in low-income communities mostly in South African rural provinces in Limpopo (Eastern Cape) and KwaZulu Natal. ^[22]^ Therefore, short- and long-term interventions need to be implemented by national government to ensure provision and access to sporting infrastructure within HDAs.

#### Lack of post-career support

Although most athletes reported receiving bursaries and scholarships, the majority highlighted poor career guidance to prepare for life after sport. Most of the athletes indicated that they had to seek opportunities during their sporting career to secure a better future after the sport. Some athletes reported losing out on beneficial time to gain valuable workplace experience as they were full-time athletes. One athlete commented as follows:

*‘To be honest, there aren't enough opportunities that propel you like a clear pathway, you create your new path to force your way through, but most of the time it's not easy.’ (Athlete 12)*

Dos Santos et al ^[23]^ showed a similar finding whereby 379 Brazilian elite athletes reported having anxiety and apprehension over the uncertainty of their future after sport as a consequence of insufficient post-career support. They indicated that this also affected their performance on the field of play. Hong and Fraser ^[24]^ reported an increased risk of mental health problems among athlete retirees who are poorly supported, especially those facing financial distress. Therefore, it is recommended that sport becomes more professionalised and that elite athletes who represent the country internationally should be presented with similar benefits as those of full-time employees, ensuring that they have access to pension fund schemes, medical insurance, and unemployment insurance funds (UIF). This will ensure that athletes have post-retirement benefits to kick-start their life after sport while seeking new opportunities. This intervention can be implemented by the government, working together with national federations. Mazzei et al. ^[2]^ who also reported insufficient post-sport career support in Brazil, suggested that a new model of athlete support needs to be established, especially in the education sector, to enable athletes to build their professional careers. Therefore, it is also advisable for athletes, in the South African context, to become student-athletes and enrol in universities or colleges through sports bursaries, to acquire qualifications and skills they can use post-retirement.

#### Poor scientific support

The majority of athletes reported having poor scientific support throughout their sporting careers. Scientific support relates to access to the latest research in training and performance, individualised training regimes, and access to sports scientists, sports medicine specialists, and other professional support structures. Some highlighted only having access to such support at a later age when they had reached elite status. Few athletes indicated that should they have had access to good scientific support throughout their career, they would have perhaps won an Olympic medal.

*‘We had no access to high-performance centres or any scientific support in the townships. In most cases there is no support in such instances unless you make the team go compete at Olympics, then in weeks before the games, you will have access to some sort of scientific testing or high-performance testing sessions.’ (Athlete 4)*

Mthombeni, Coopoo, and Noorbhai ^[25]^ reported similar findings when evaluating the availability of support systems by South African national sport federations, whereby majority reported a lack of scientific support programmes within their administration. Sports science research and scientific support leads to evidence-based training regimes that allow athletes to perform optimally. This is more important for an individual-specific scientific-backed programme and training. Poor scientific support to athletes often leads to them falling victim to pseudoscientific practices, including the use of unproven training techniques, the wastage of money on supplements that do not improve their athletic ability, and other misleading practices that sports people fall victim to. With evolving technology, sport has become more competitive and scientific. Sports scientists and medical professionals can now leverage application software, social media, and other forms of technological interventions to assist athletes in achieving optimal performance. This makes scientific support more important now, than ever before. One of the studies by Kubayi, Coopoo, and Toriola ^[26]^ found that coaches have previously reported that the most important priorities in providing scientific support to athletes were technique improvement, injury prevention, peaking for competition, as well as mental strength. Therefore, it is important to bridge a gap between sports science support and coaching to ensure that coaches have access to evidence-based regimes to impart to athletes.

### Limitations

The limitation of the study was that it was only qualitative and had to rely on subjective data from a selected cohort of athletes. However, the qualitative method was also a strength of the study in that it provided rich in-depth and lived experiences of athletes. Another limitation was that the participants were all black African athletes, limiting the diversity of views from different racial groups. Future studies should focus on a quantitative model of measuring elite athlete support systems. The study only focused on collecting data from former Olympians and did not focus on the current developmental athletes which would indulge in the challenges they are facing currently.

## Conclusion

The study showed that South African athletes from HDAs attributed their sporting success to the functional club system sport structure, access to sports at the community level, access to scholarships and bursaries to elite schools or universities, good coaching support and mentorship, access to local and international competitions, as well as community and peer athlete support. The highest barriers reported by athletes were inadequate financial support (especially during the developmental stages of their career), a dysfunctional school sports system, lack of sports facilities, equipment and transport system, poor post-career support, as well as lack of scientific support. Financial investment in elite sports is necessary for the country to achieve its full potential for international success. Developing (up-and-coming) athletes from HDAs need to be financially supported consistently through athlete-centred support programmes to ensure that they have access to the necessary resources for them to excel in their respective sporting codes. The government should ensure that organised sport becomes a compulsory component of the schooling system across all South African schools. The national government should take on the responsibility for the provision of adequate sports facilities in HDAs. Elite athletes who have represented South Africa internationally should be protected under the nation's labour laws to ensure that they have similar benefits to ordinary employees for them to enjoy social protection. There needs to be a bridging of the knowledge gap between coaches and sports scientists to allow coaches to use evidence-based approaches in athletic training and programming.

Future studies need to focus on in-depth analysis of evaluating the impact of class, socioeconomic status, gender and the impact of such variables in providing access, participation, and success in sports. Future studies should also provide a sport-specific analysis to assess which sports are more accessible than others by athletes from HDAs and factors that may be enablers or hindrances to participation in specific sporting codes.

## Figures and Tables

**Fig. 1:**
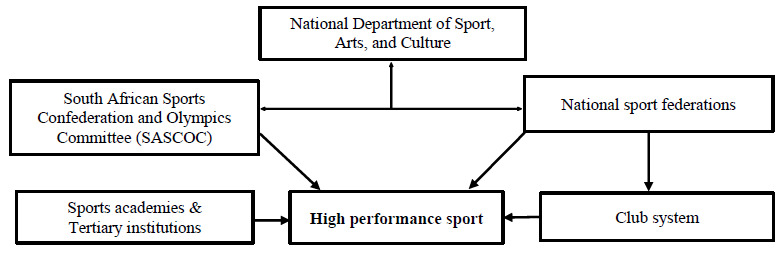
South African support mechanism for high performance sport ^[4]^

**Table 1:** Participant demographics

Participant	Gender	Race	Age (years)	Sport	Number of years of involvement
**A1**	Male	Black	47	Athletics - Track	37
**A2**	Male	Black	50	Athletic - Marathon	28
**A3**	Male	Black	48	Athletics - Track	32
**A4**	Male	Black	45	Athletic - Marathon	32
**A5**	Male	Black	37	Athletic - Marathon	21
**A6**	Female	Black	39	Athletic - Marathon	33
**A7**	Male	Black	47	Athletic - Marathon	29
**A8**	Male	Black	30	Boxing	18
**A9**	Male	Black	44	Boxing	37
**A10**	Male	Black	32	Rugby Sevens	20
**A11**	Male	Black	29	Rugby Sevens	19
**A12**	Female	Black	34	Soccer	24
**A13**	Male	Black	38	Canoeing	27
**A14**	Male	Black	30	Sailing	19
**A15**	Male	Black	38	Hockey	25

**Table 2:** Explanatory notes for the nine pillars used in the questionnaire

Pillar	Explanatory notes for the pillar
**1. Financial resources**	This includes financial resource assistance in the form of grants (from government, federations, NGOs, sponsorships, etc.).
**2. Organisation and the structure of sport policies**	Refers to the availability and access to the sport competition system at all levels, including local, provincial and national level.
**3. Foundation and participation**	Refers to the accessibility and participation of the sport code at foundation phase (i.e. school competition system, youth club/development academy, etc.).
**4. Talent identification and development system**	Refers to the systems in place to identify and develop sporting talent (i.e. talent scouting, sport career pathing, etc.).
**5. Post-career support**	Refers to support provided to athletes upon retirement from their sport (scholarships and bursaries, life skills, career/job prospects, mentoring opportunities, volunteerism, etc.) or any other opportunity to make a living after sport.
**6. Sports training facilities**	Refers to the availability of sports facilities that athletes have access to.
**7. Coach provision and development**	Refers to availability and access to qualified/experienced coaches.
**8. Local and international competitions**	Refers to availability and access to local and international competitions where athletes can compete and showcase their talent.
**9. Scientific support**	Refers to availability and access to scientific support programmes for the athlete (i.e. sports medicine/science, evidence-based programmes and research, nutrition, sports psychology, high performance, match/performance analysis, etc.).

Sourced from Sport Policy Factors Leading to Sport Success (SPLISS) framework ^[1]^
